# Pulmonary Embolism Revealing Idiopathic Membranous Glomerulonephritis

**DOI:** 10.1155/2010/683652

**Published:** 2010-09-20

**Authors:** A. Hamzaoui, O. Harzallah, R. Klii, L. Njim, S. Mahjoub

**Affiliations:** ^1^Departement of Internal Medicine, Fattouma Bourguiba Hospital, 5000 Monastir, Tunisia; ^2^Department of Anatomopathology, Fattouma Bourguiba Hospital, 5000 Monastir, Tunisia

## Abstract

We describe a case of a 55-year-old man who presented with pulmonary embolism and who was found to have nephrotic syndrome due to idiopathic membranous nephropathy. There are no other signs of nephrotic syndrome such as edema.

## 1. Introduction

The nephrotic syndrome is a risk factor for venous thrombosis wether it is strongly associated with hypercoagulability and alteration in various coagulation factors. Pulmonary embolism in patients with nephrotic syndrome is caused by deep venous thrombosis or renal venous thrombosis. The prevalence of pulmonary embolism in patient with the nephrotic syndrome is based on case series.

We report the case of a 55-year-old man who presented with pulmonary embolism and was subsequently found to have asymptomatic nephrotic syndrome due to idiopathic membranous nephropathy.

## 2. Case Report

A 55-year-old man presented with chest pain. There was no swelling in his legs. On physical examination, he was apyrexic, with a respiratory rate of 34 breaths per minute, an oxygen saturation of 96% in room air, a heart rate of 128 beats per minute and arterial blood pressure of 130/80. The heart sounds were regular, with no added murmurs, and auscultation of the lungs was normal. There was no edema in the legs. The findings on the neurological, musculoskeletal and skin examinations were normal. The electrocardiogram showed sinus tachycardia and no other abnormalities. The arteriel blood gases were normal, with a pH of 7,44, partial pressure of oxygen (Pa O2) of 90 mmHg, partial pressure of carbon dioxide (Pa CO2) of 38 mmHg, and bicarbonate of 24 mmo/l in room air. Doppler ultrasonography was normal. D-Dimers were high (>4000 ng/l). The chest radiography was normal. Thoracic tomography showed massive bilateral proximal thrombosis of the pulmonary arteries. Moderate pulmonary hypertension was found on cardiac echography (PAP = 35 mmHg).

Acute pulmonary embolism was diagnosed. The patient was initially treated with enoxaparin (80 mg twice per day) and then with Sintrom*. Other sites of thrombosis were excluded by Doppler sonography of the lower limb and renal veins.

Thrombophilia screening tests, including: Protein C and S, Antithrombin III, homocysteine level, factor V mutations, and antiphospholipid antibodies, were normal. Renal function was normal with serum creatinine 89 *μ*mol/l. Urine protein was quantified at 10 g/24 h. Serum protid and albumin were respectively of 51 and 17 g/l. A diagnosis of nephrotic syndrome was confirmed. The total cholesterol was of 6,75 mmol/l and triglyceride of 2,11 mmol/l.

No renal venous thrombosis was found on the Doppler sonography.

**Figure 1:**
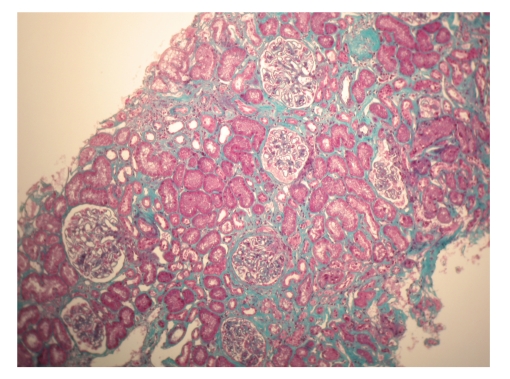
Tubulointerstial fibrosis with thickened glomerular basement membrane (Masson's trichrome stain, x200).

**Figure 2:**
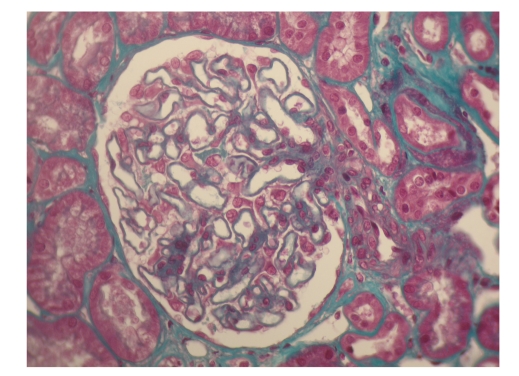
Glomeruli with thickened basement membrane (Masson's trichrome, x400).

Renal biopsy showed that this was caused by membranous nephropathy. It showed glomeruli with mild thickening of the capillary walls with patent capillary loops. There was no mesangial hypercellularity and the tubules, interstitium and vessels were normal. Immunofluorescence showed strong granular Ig G along the capillary wall and some complement. No Ig A deposition was seen (Figures 1 and 2). 

Our patient received no drugs; no features of acute or chronic infections were found. The investigating for secondary causes of this membranous glomerulonephritis included: thoracoabdominal tomography, tumor markers, thyroid hormone, and endobrachial endoscopy were normal.

Treatment was based on the alternating pulse of methylprednisolone, oral corticosteroid and cyclophosphamide. Enalapril (10 mg per day) and statin were prescribed. At 2 months of treatment, the proteinuria decreased at 3 g/24 h.

## 3. Discussion

Pulmonary embolism is a rare but potentially life threatening complication of nephrotic syndrome.

In patient with nephrotic syndrome, there is evidence of clotting factors such as fibrinogen, factors V and VIII, von Willebrand factor, and plasminogen activator inhibitor 1. There is also an increasing urinary loss of inhibitors of coagulation (Antithrombin III, plasminogen) [1]. We also reported low levels of anti thrombin III in renal vein, increased in platelet aggregation and inherited factor V Leiden deficiency [2].

A review of eight studies evaluating thromboembolic complications in nephrotic syndrome found 81 (18%) of 458 patients with deep venous thromboembolism or pulmonary embolism [3]. The incidence varied from 8%, 5% to 36% in the literature. The renal venous thrombosis is the most frequently detected; it is asymptomatic in 90% of cases. In children, pulmonary embolism is the second most frequent thromboembolic complication in nephrotic syndrome and most cases are secondary to renal venous thrombosis [4].

A recent clinical study research including 925.000 patients from 1979 to 2005 with the diagnosis of nephrotic syndrome found: 5000 pulmonary embolism (0.5%), 14000 deep venous thrombosis (1.25%) and fewer than 5000 patients with renal venous thrombosis. When comparing to 893.253.000 hospitalized patients without nephrotic syndrome, the relative risk of pulmonary embolism was 1,39 and of deep venous thromboembolism was of 1,72 [5].

We report a case of pulmonary embolism revealing nephrotic syndrome due to idiopathic membranous nephropathy. Our patient presented 2 risk factors for thrombosis: A serum albumin <20 g/l and a proteinuria above 3 g/24 h.

The association of membranous nephropathy and renal vein thrombosis is more known than any other venous thrombosis, but it can occur in any site and with other causes of nephrotic syndrome. In our case, pulmonary embolism was the first presenting feature of the nephrotic syndrome. It was even reported by Hartland et al. [6], Peces et al. [7] and Ambler et al. [8]. No other site of thrombosis was detected in our patient.

Among 32 patients with nephrotic syndrome, 4 (12. 5%) were found to have presented with deep venous thrombosis [2] or pulmonary embolism [2]. Three had membranous nephropathy and one had minimal change nephropathy. Renal vein Doppler studies were normal in both patients with pulmonary embolism, like our patient [8].

Pulmonary embolism may also complicate the course of a known membranous glomerulonephritis, especially when the disease is still active. Kutcher and coll described 2 cases of known membranous glomerulonephritis complicated with renal veins thrombosis and pulmonary embolism [9]. A recurrence of pulmonary embolism with renal veins and inferior vena cavography thrombosis characterized the case of Briefel and coll [10]. The same clinical finding was described by Pasquariello and Camerini [11], Min et al. [12], and Irie et al. [13]; renal veins thrombosis was associated in all this cases.

Recently, Harroche described a case of pulmonary embolism revealing a systemic lupus erythematosus with membranous glomerulonephritis in a 14-year-old boy [14].

As known, glomerulonephritis is the major cause of nephrotic syndrome and membranous glomerulonephritis represent 40% of the nephrotic syndrome in adolescent and young adult. It is idiopathic in over two-thirds of adult [15], like in our patient.

We add to that the fact that membranous glomerulonephritis is inherently thrombogenic for understood reasons; the thromboembolic complications seems to be more frequent when the nephrotic syndrome is due to membranous nephropathy than other causes [1].

Membranous glomerulonephritis can be primary (idiopathic) or secondary to drugs (such as nonsteroidal antiinflammatory drugs, gold, penicillamine), malignancy (breast or bronchial carcinoma), infection (particularly immunodificiency virus, hepatitis B or C virus infection, systemic disorder (systemic lupus erythematosus), or hypothyroidism [16]. No secondary cause was found in our case.

Approximately 25% of cases of membranous glomerulonephritis resolve spontaneously. The indications for and types of treatment are controversial. There is no good evidence in favor of therapies based on corticosteroids alone. Cyclophosphamide and chlorambucil may increase the probability of remission. Good results have been obtained by alternating corticosteroids and a cytotoxic agent every other month for 6 months. Other molecul may be prescribed: cyclosporine, mycophenolate mofetil, rituximab and immunoglobulins intravenous [15]. In 30% of patients, a progressive renal failure can occur, despite immunosuppression [17]. Our patient received alternating pulse methylprednisolone, oral corticosteroid, and cyclophosphamide with good response.

Further studies might allow us to better understand the physiopathology of MN and to organize more specific and effective treatments in the near future.

## 4. Conclusion

Nephrotic syndrome is complicated by venous thromboembolism sufficiently frequently for the diagnosis to be considered in all patients with deep venous thromboembolism or pulmonary embolism. The message is simply don't forget to dip the urine and the serum albumin in this patients.
